# A novel FC17/CESA4 mutation causes increased biomass saccharification and lodging resistance by remodeling cell wall in rice

**DOI:** 10.1186/s13068-018-1298-2

**Published:** 2018-11-01

**Authors:** Fengcheng Li, Sitong Liu, Hai Xu, Quan Xu

**Affiliations:** Rice Research Institute, Shenyang Agricultural University, Shenyang, 110866 China

**Keywords:** Biomass saccharification, Cell wall, Cellulose CrI, CESA, Lodging resistance, Rice

## Abstract

**Background:**

Rice not only produces grains for human beings, but also provides large amounts of lignocellulose residues, which recently highlighted as feedstock for biofuel production. Genetic modification of plant cell walls can potentially enhance biomass saccharification; however, it remains a challenge to maintain a normal growth with enhanced lodging resistance in rice.

**Results:**

In this study, rice (*Oryza sativa*) mutant *fc17*, which harbors the substitution (F426S) at the plant-conserved region (P-CR) of cellulose synthase 4 (CESA4) protein, exhibited slightly affected plant growth and 17% higher lodging resistance compared to the wild-type. More importantly, the mutant showed a 1.68-fold enhancement in biomass saccharification efficiency. Cell wall composition analysis showed a reduction in secondary wall thickness and cellulose content, and compensatory increase in hemicelluloses and lignin content. Both X-ray diffraction and calcofluor staining demonstrated a significant reduction in cellulose crystallinity, which should be a key factor for its high saccharification. Proteomic profiling of wild-type and *fc17* plants further indicated a possible mechanism by which mutation induces cellulose deposition and cell wall remodeling.

**Conclusion:**

These results suggest that CESA4 P-CR site mutation affects cell wall features especially cellulose structure and thereby causes enhancement in biomass digestion and lodging resistance. Therefore, CESA4 P-CR region is promising target for cell wall modification to facilitate the breeding of bioenergy rice.

**Electronic supplementary material:**

The online version of this article (10.1186/s13068-018-1298-2) contains supplementary material, which is available to authorized users.

## Background

Plant cell walls are essential for plant growth and development and provide renewable biomass feedstock for biofuels production [1]. The recalcitrance of plant cell walls leads to a costly biomass process. To reduce recalcitrance, genetic modifications of wall polymers have been applied to enhance biomass saccharification [2–4]. However, alterations in wall polymers are mostly associated with defects in plant growth and development; it becomes critical to identify a key gene for cell wall modification that could not substantially affect plant growth but leads to an enhancement in biomass saccharification [5].

Plants typically contain two different types of cell walls: namely, primary cell walls (PCWs) and secondary cell walls (SCWs). During growth, plant cells are surrounded by a strong yet adaptable primary cell wall, which is mainly composed of cellulose, hemicelluloses, pectins, and proteins [6]. Once growth has ceased, an additional secondary wall may be deposited, which is mainly composed of cellulose, hemicelluloses, and lignin. Cellulose, consisting of linear chains of β-1,4-linked glucan, is the principal substrate for bioethanol production. Through intra- and inter-chain hydrogen bonding, parallel linear glucan chains are crystalized to form cellulose microfibrils that provide plants with excellent toughness for normal plant growth [1]. Crystallinity index (CrI) is a parameter commonly used to quantify cellulose microfibrils crystallinity [7–9], which is one of the most important characteristics of cellulose negatively affecting biomass enzymatic hydrolysis [10–13]. Hemicelluloses are a heterogeneous class of polysaccharides with various sugar units, and arabinoxylans comprise the majority of hemicelluloses in the mature tissues of rice [10, 14]. Lignin is a complex phenolic polymer that provide plants with great stiffness for resistance of biotic or abiotic stress [15, 16].

Cellulose biosynthesis is a tightly regulated process that is performed by plasma membrane-spanning cellulose synthase (CESA) complexes (CSCs) [1]. The CESA protein in different plant species shares common domains and motifs, such as the zinc fingers, the central cytoplasmic domain with D,D,D,QXXRW motif, and eight transmembrane domains [17, 18]. The central cytoplasmic domain contains the plant-conserved region (P-CR) and class-specific region (CSR), which may play a role in CESA protein association and assembly [19, 20]. Genetic and biochemical studies suggest that at least three distinct CESA isoforms are required to form functional CSCs [21]. In *Arabidopsis thaliana*, CesA1, CesA3, and CesA6-like (i.e., CesA2, 5, 6, and 9) CesAs synthesize primary wall cellulose, and CesA4, CesA7, and CesA8 comprise the CSCs necessary for secondary wall cellulose production [21–23]. Bioinformatics and mutational analysis have shown that OsCesA1, 3, 8 and OsCesA4, 7, 9 are involved in the cellulose synthesis of the primary and secondary cell walls in rice, respectively [24–26].

Up to date, three *OsCesA4* mutants have been identified in rice, and all of them show brittleness due to reductions of cellulose content [26–28]. Apart from the brittleness phenotype, *Tos17* insertional mutant of CESA4 displays the semi-dwarfism and withering of leaf apex [26]. Bc7 (t) has a two-base deletion in exon 10 and a five-base deletion in intron 10 and shows indistinguishable phenotypes from the wild-type plants [28]. Different from BC7, BC11 mutation occurs in the fifth transmembrane domain of CESA4 and causes severe dwarfism and low pollen fertility [27]. These studies demonstrate that mutations in different sites of CESA4 lead to diverse effects on plant growth, indicating a complex role of CESA4 in plant morphological development.

Rice is a major global food crop that produces enormous biomass residues for biofuels and chemical products [29]. In this study, we report on a novel CESA4 conserved site mutation that leads to increased lignocellulose enzymatic hydrolysis and lodging resistance by reducing cellulose CrI and remodeling cell wall.

## Results

### The *fc17* mutant has a brittleness phenotype caused by a CESA4 conserved site mutation

**Fig. 1:**
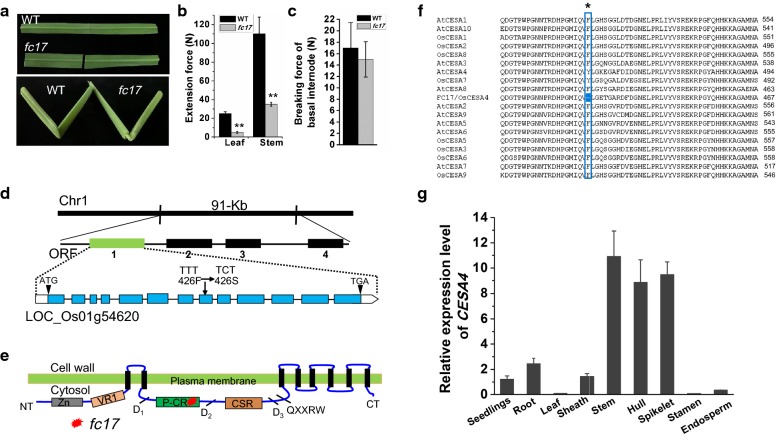
Phenotypic observations and map-based cloning of the gene for *fc17*. **a** Brittleness of leaves and culms. **b** Measurements of the extension forces (Newtons) of leaves and culms. **c** Measurements of the breaking forces (Newtons) of basal stem internodes. **d** Location of *fc17* mutation with substitutions of Phe residues with Ser at the 426th position of the CESA4 protein. **e** Schematic diagram of domains and the location of *fc17* mutation in CESA4. **f** Protein sequences alignment of FC17 protein family. Black asterisk represents the mutated amino acid. **g** Expression pattern of *CESA4* in various tissues and organs

A *fragile culm 17* (*fc17*) mutant was isolated from a natural population of the *Japonica* cultivar *ShenNong265* (Fig. 1a). The extension forces of culms and leaves in *fc17* were respectively reduced to ~ 20% and ~ 30% of those in the wild-type (WT) (Fig. 1b). Despite brittleness and reduced extension force, *fc17* did not show significant reduction in breaking force of basal stem internode, an important physiological properties associated with plant lodging resistance (Fig. 1c).

**Fig. 2:**
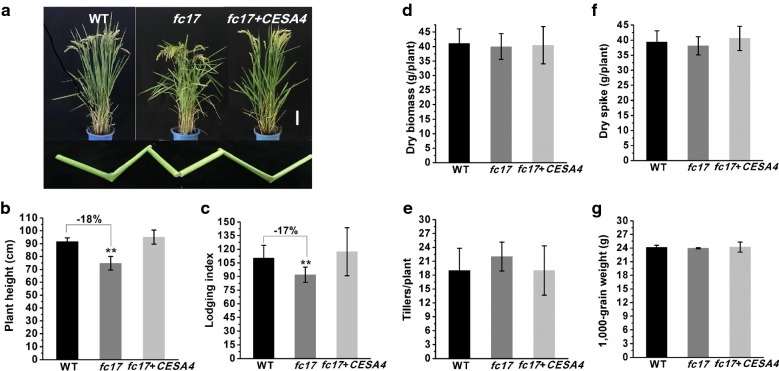
Agronomic traits observation. **a** Phenotypes and physical properties of the wild-type (WT), *fc17* mutant, and complementary lines (*fc17* + *CESA4*) at the mature stage (scale bar = 10 cm). **b** Plant height. **c** Lodging index. **d** Dry biomass. **e** Tiller numbers. **f** Dry spike. **g** 1000-grain weight. **Indicate significant differences between WT and *fc17* mutant by *t*-test at *p* < 0.01, with the increased or decreased percentage (%) calculated by subtraction of the values between the mutant and WT and divided by WT. The error bar indicates SD values (*n* = 3)

Using 950 F_2_ mutant plants generated from a cross between *fc17* and an *indica* cultivar *MH63*, we identified via map-based cloning a 91-kb region on chromosome 1 that contains the candidate gene. The region includes four putative candidate genes. The first putative is ORF1 (TIGR ID: LOC_Os01g54620), which encodes cellulose synthase catalytic subunit 4 (*OsCESA4*), involving in secondary wall cellulose biosynthesis (Fig. 1d). Sequencing analysis revealed a single-base substitution at the eighth exon, changing TTT to TCT, and resulting in a change in the 426th amino acid from Phe to Ser (Fig. 1d). This substitution occurred at the P-CR region (Fig. 1e), which is conserved in all CESA family proteins of rice and *Arabidopsis* (Fig. 1f). Quantitative PCR analysis of CESA4 in multiple tissues and organs revealed that CESA4 primarily expressed in the secondary wall-rich tissues (stem, hull and spikelet) (Fig. 1g). The *fc17* phenotypes were further complemented by transgenic expression of WT *CESA4* in the *fc17* mutant background (*fc17* + *CESA4*), confirming that the missense mutation of CESA4 was responsible for the mutant phenotypes (Fig. 2).

### The *fc17* mutant exhibits a normal plant growth with higher lodging resistance

Apart from brittleness, the *fc17* mutant exhibited a normal plant growth, similar to that in the WT (Fig. 2a). Despite the relatively short height (Fig. 2b), 2-year field experiments showed that the *fc17* mutant had significantly improved plant lodging resistance (lodging index was reduced by 17%) (Fig. 2c; Additional file 1). In addition, no significant differences in other important agronomic traits of the WT and *fc17*, including dry biomass, tiller number, dry spike, and 1000-grain weight, were observed (Fig. 2d–g).

### The *fc17* mutant displays enhanced biomass enzymatic saccharification

**Fig. 3:**
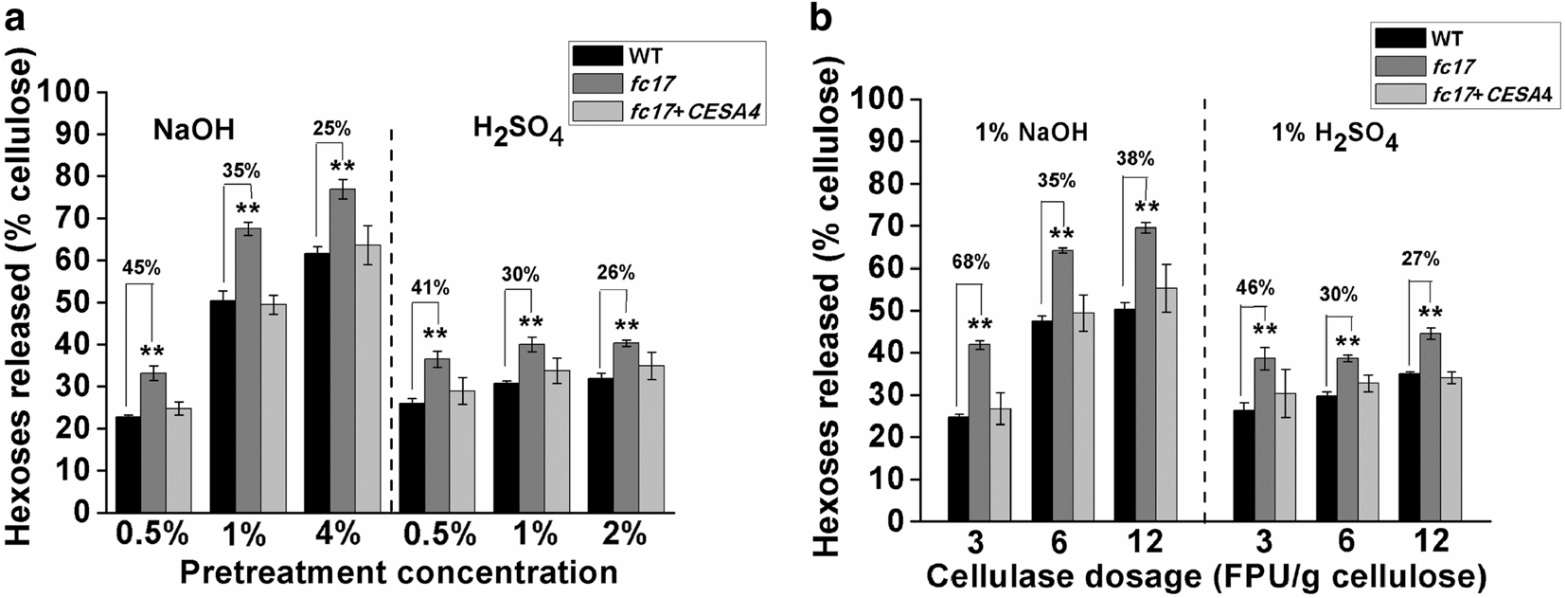
Biomass enzymatic saccharification of the wild-type (WT) and *fc17* plants. **a** Hexose yields released from enzymatic (mixed-cellulase) hydrolysis after pretreatment with NaOH and H_2_SO_4_ at three concentrations. **b** Hexose yields released from three dosages of mixed-cellulase hydrolysis after pretreatment with 1% NaOH and 1% H_2_SO_4_. **Indicate significant differences between WT and *fc17* mutant by *t*-test at *p* < 0.01, with the increased percentage (%) calculated by subtraction of the values between mutant and WT and divided by WT. The error bar indicates SD values (*n* = 3)

Using mature stem materials, we measured biomass enzymatic digestibility (saccharification) in the *fc17* mutant by calculating the hexose yields released from enzymatic hydrolysis of pretreated biomass. The *fc17* mutant exhibited significantly higher hexoses yields by up to 1.45-fold than that of the WT, with pretreatments using three concentrations of alkali (0.5%, 1%, and 4% NaOH) and acid (0.5%, 1%, and 2% H_2_SO_4_) (Fig. 3a).

To evaluate the effect of enzyme dosage on the saccharification of *fc17* and WT plants, enzymatic hydrolysis was performed with different cellulase loadings of 3, 6, and 12 filter paper unit (FPU) per gram of cellulose. A more efficient conversion of lignocellulosic biomass to hexose was observed in the *fc17* mutants, which was up to 1.68-fold higher than that of WT plants (Fig. 3b).

**Fig. 4:**
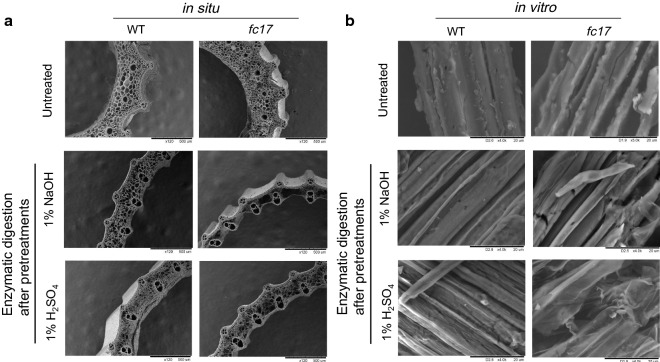
Observation of biomass digestion. **a** Scanning electron microscopy (SEM) images of in situ enzymatic digestion of stems at heading stage after 1% NaOH or 1% H_2_SO_4_ pretreatment and sequential enzymatic hydrolysis. **b** SEM images of in vitro enzymatic digestion of biomass residues released from enzymatic hydrolysis after 1% NaOH or 1% H_2_SO_4_ pretreatment

To confirm the enhanced biomass enzymatic digestibility of the *fc17* mutant, we evaluated their stem cell tissues in situ and biomass residues in vitro under SEM (Fig. 4). Without any pretreatment and enzymatic digestion, no visible in situ differences between stems of the mutant and WT were observed (Fig. 4a). However, the mutant exhibited more severe destruction and cell loss than that of the WT after 1% NaOH or 1% H_2_SO_4_ pretreatment and sequential enzymatic hydrolysis (Fig. 4a). In addition, the biomass residues of the mutant showed rougher surfaces in vitro compared to the WT under 1% NaOH or 1% H_2_SO_4_ pretreatments and sequential enzymatic hydrolysis (Fig. 4b), which agrees with the findings in other grass plants [11, 30]. Both in situ and in vitro observations thus confirm that the mutant has high biomass enzymatic digestibility.

### The *fc17* mutant shows altered cell wall structure and composition

**Fig. 5:**
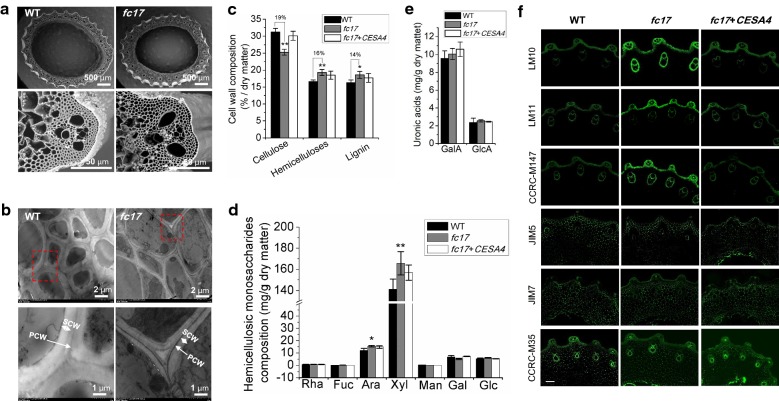
Observations of stem tissues and cell wall structures. **a** Scanning electron microscopy (SEM) images of the second-internode stem at the heading stage of rice. **b** Transmission electron microscopy (TEM) images of the sclerenchyma cell walls. *PCW* primary cell wall, *SCW* secondary cell wall. **c** Cell wall polymer determination. **d** Hemicellulosic monosaccharides composition. **e** Determination of galacturonic acid (GalA) and glucuronic acid (GlcA). **f** Immunodetection of transverse sections of *fc17* and wild-type (WT) culm internodes. Representative micrographs of equivalent sections of mature culm internodes immunolabeled with antibodies directed to xylan (LM10, LM11, and CCRC-M147), homogalacturonan (HG; JIM5 and JIM7) and rhamnogalacturonan-I (RG-1; CCRC-M35) (scale bar = 100 μm). *, ** Indicated significant differences between the WT and *fc17* mutant by *t*-test at *p* < 0.05 and 0.01, respectively, with the increased percentage (%) calculated by subtraction of the values between mutant and WT and divided by WT. The error bar indicates SD values (*n* = 3)

To understand the improved lodging resistance and enhanced biomass digestibility in the *fc17* mutant, we examined its cell wall structure and composition. Scanning electron microscopy (SEM) showed that the *fc17* had no alterations in the culm cross section structure and cell size, but exhibited reduced wall thickness (Fig. 5a). Transmission electron microscopy (TEM) further revealed that the *fc17* plants displayed thinner and uneven secondary cell walls compared to the WT plants (Fig. 5b).

To investigate the underlying cause of the altered cell wall structure in *fc17* plants, we performed chemical measurement of three wall polymers in secondary cell wall-rich stems of *fc17* and WT plants. The *fc17* mutant showed a 19% reduction in cellulose levels and a 16% increase in hemicelluloses level and a 14% increase lignin level compared to that in the WT (Fig. 5c). Furthermore, GC–MS analyses revealed that the increase of hemicelluloses content in *fc17* was mainly contributed by the increase in hemicellulosic arabinose and xylose levels (Fig. 5d). In addition, the *fc17* exhibited similar content of galacturonic acid (GalA) and glucuronic acid (GlcA) as compared with WT (Fig. 5e).

To confirm the wall polymer content detected by chemical approach, we compared equivalent transverse sections of *fc17* and WT culm internodes labeled with antibodies against xylan (LM10, LM11, and CCRC-M147), homogalacturonan (HG; JIM5 and JIM7) and rhamnogalacturonan-I (RG-I; CCRC-M35). All antibodies directed to xylan showed a significantly increased occurrence of their epitope in *fc17*, while HG and RG-1 did not show significant difference between the *fc17* and WT plants (Fig. 5f).

### The *fc17* mutant produces structurally aberrant cellulose microfibrils

**Fig. 6:**
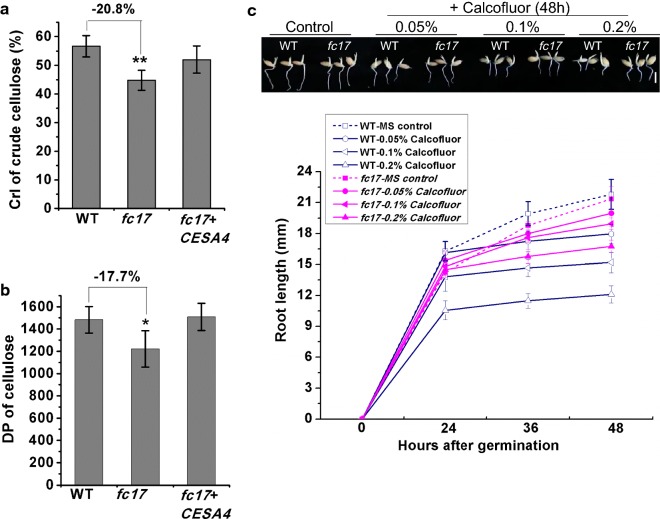
Detection of cellulose structural features. **a** Lignocellulose CrI of mature stems using the X-ray diffraction (XRD) method. **b** Cellulose DP of mature stems using viscometry method. **c** Root lengths of the germinated seedlings treated with Calcofluor for 48 h (scale bar = 1 cm). *, **Indicate significant differences between the wild-type (WT) and *fc17* mutant by *t*-test at *p* < 0.05 and 0.01, respectively, with the decreased percentage (%) calculated by subtraction of the values between mutant and WT and divided by WT. The error bar indicates SD values (*n* = 3)

Mutations involving CESA proteins not only affect cellulose level but also influence cellulose structure features [31, 32]. To explain the increased biomass saccharification in *fc17*, we measured the cellulose crystallinity of both the WT and mutant using the mature stem. X-ray diffraction analysis estimated a 20.8% reduction in cellulose crystallinity in the *fc17* mutant compared to the WT (Fig. 6a). As cellulose CrI is positively correlated with its degree of polymerization (DP) [12, 24], we further examined the cellulose DP of mature stem in both the WT and mutant plants. A 17.7% reduction in the cellulose DP was observed in the *fc17* mutant compared to the WT, suggesting that this may have contributed to the observed lower CrI (Fig. 6b).

Cellulose crystallization can be prevented using dyes that interact with cellulose chains such as Calcofluor White [33]. To confirm the observed decrease in cellulose CrI in *fc17* in vitro, we treated the mutant and WT seedlings after germination with three concentrations of calcofluor and then measured the lengths of their root. Calcofluor dramatically repressed root growth in the WT using the intermediate (0.1%) concentration and completely prevented root elongation using a high (0.2%) concentration (Fig. 6c). However, the *fc17* mutant was highly resistant to calcofluor even at the highest concentration (0.2%) (Fig. 6c). The observed greater resistance of *fc17* to high-concentration calcofluor demonstrates that its root growth was insensitive to calcofluor (Fig. 6c). The differences in the response of *fc17* and WT to calcofluor support that *fc17* possesses an altered cellulose microfibril structure.

### Comparative proteomic analysis of WT and *fc17* plants

**Fig. 7:**
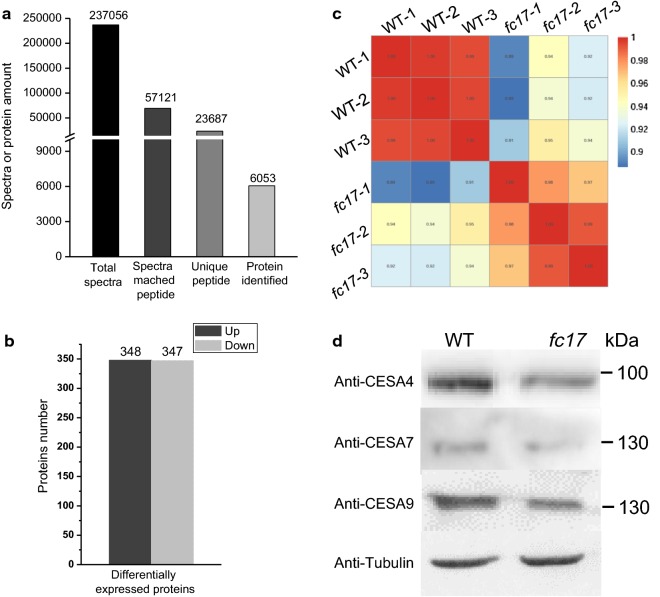
Proteome profile analysis of wild-type (WT) and *fc17* mutant by iTRAQ-based proteomic approach. **a** Results of mass spectrometry analysis and protein identification. **b** Number of proteins that are upregulated and downregulated in *fc17* compared with those in the WT. **c** Pearson correlation analysis among the samples from different biological replicate. **d** Western blot analysis of CESA4, CESA7, and CESA9 using specific antibody

To elucidate the molecular mechanism(s) by which FC17 regulates the cell wall formation and maintains plant growth, we used an iTRAQ-based (isobaric tags for relative and absolute quantification) proteomics analysis of rice stems at heading stage to identify changes in protein expression between the WT and *fc17* plants. After iTRAQ and liquid chromatography–tandem mass spectrometry (LC–MS/MS) analysis, a total of 237,056 spectra were identified, of which 57,121 spectra could be matched to peptides in the database and 23,687 were unique peptides (Fig. 7a). A total of 6053 proteins were identified using the *Oryza sativa* UniProt database. We detected 695 differentially expressed proteins (DEPs) with expression changes > 1.5-fold between the WT and *fc17*. Of these proteins, 348 were upregulated and 347 were downregulated (Fig. 7b). Pearson correlation analysis showed good correlation among different replicates of the same sample, whereas significant differences were observed between the WT and *fc17* plants (Fig. 7c).

Based on the considerable effects of the *fc17* mutation on cellulose content and CrI, we quantified the expression levels of proteins involved in cellulose production processes by iTRAQ proteasome profile. The protein levels of CESA4, CESA7, and CESA9 forming the secondary wall CSC were respectively reduced by 18%, 15%, and 11% in *fc17* (Table 1), which was confirmed by western blot analysis of CESA4, CESA7, and CESA9 using specific antibody (Fig. 7d). In contrast, the other CESA proteins (CESA1/2/3/6/8) in *fc17* plants displayed similar expression level compared to that in the WT (Table 1). We also examined the expression levels of BC (Brittle Culm) proteins which contribute to both mechanical strength and cellulose synthesis in rice (Table 1). Most of the BC proteins quantified by iTRAQ assay did not exhibited significant alterations between *fc17* and WT plants, with the exception of BC12 (KIF4 family protein) which was reduced by 12% in the *fc17* mutant (Table 1). Therefore, the *fc17* mutation probably regulates the cellulose production through impairing CESA subunits numbers or association of the secondary cell wall CSCs.

**Table 1:** Expression levels of CESA and BC (Brittle Culm) proteins in the wild-type (WT) and *fc17* plants by iTRAQ analysis

Name	Locus	WT	*fc17*	*fc17*/WT ratio	*p*	Change
CESA4	LOC_Os01g54620	122.87	100.5	0.82	0.003	Down
CESA7	LOC_Os10g32980	135.27	115.2	0.85	0.001	Down
CESA9	LOC_Os09g25490	111.27	99.57	0.89	0.016	Down
CESA1	LOC_Os05g08370	2783.43	2965.9	1.07	0.622	
CESA2	LOC_Os03g59340	420.27	410.33	0.98	0.084	
CESA3	LOC_Os07g24190	1978.77	1949.47	0.99	0.888	
CESA6	LOC_Os07g14850	386.27	409.43	1.06	0.126	
CESA8	LOC_Os07g10770	1506.37	1607.2	1.07	0.626	
BC3	LOC_Os02g50550	14299.03	13301.97	0.93	0.067	
BC10	LOC_Os05g07790	1273.9	1179.7	0.92	0.052	
BC12	LOC_Os09g02650	71.5	63.4	0.88	0.001	Down
BC15	LOC_Os09g32080	1211.83	1244.4	1.03	0.263	

We further employed Kyoto Encyclopedia of Genes and Genomes (KEGG) enrichment analysis to reveal the pathway affected by the *fc17* mutation (Table 2). Although the detected DEPs may be involved in 83 pathways, the categories of carbon fixation and metabolism (ko00710, ko01200), starch and sucrose metabolism (ko00500), phenylpropanoid biosynthesis (ko00940), and phenylalanine metabolism (ko00360) were apparently affected (Table 2). Most of lignin biosynthesis-associated proteins that are involved in the phenylpropanoid biosynthesis and phenylalanine metabolism pathways were upregulated in *fc17* (Additional files 2, 3). This result coincides with the higher lignin content in *fc17* relative to the WT. All of 54 DEPs that exist in both carbon fixation and carbon metabolism pathways were upregulated in *fc17* (Additional files 4, 5). In the starch and sucrose metabolism pathway, 12 of the 16 DEPs annotated as glycosyl hydrolase, sucrose synthase and glucanotransferase were upregulated in *fc17* (Additional file 6). These proteins produce more substrate for biosynthesis of hemicelluloses and other polysaccharides and might participate in hemicelluloses modification. In addition, two proteins IRX10 (LOC_Os01g70200) and IRX14 (LOC_Os06g47340) which have been reported to be involved in xyan biosynthesis were upregulated in *fc17* (Additional file 7) [34, 35]. These results coincides with the higher hemicelluloses content in *fc17* relative to the WT. These findings suggest that the *fc17* mutation probably triggers feedback responses to the cell wall defects, which in turn regulate the cell wall for the maintenance of plant growth and development.

**Table 2:** The altered pathways identified by DEPs between the wild-type (WT) and *fc17* plants using KEGG enrichment analysis

Pathway	DEPs with pathway annotation	All proteins with annotation pathway	*p*	*q*	Pathway identifier
Photosynthesis	27 (9.57%)	44 (1.98%)	1.89E−14	1.55E−12	ko00195
Phenylpropanoid biosynthesis	29 (10.28%)	82 (3.7%)	6.37E−08	2.61E−06	ko00940
Phenylalanine metabolism	12 (4.26%)	24 (1.08%)	9.29E−06	1.90E−04	ko00360
Carbon fixation	19 (6.74%)	62 (2.79%)	1.32E−04	2.16E−03	ko00710
Starch and sucrose metabolism	16 (5.67%)	73 (3.29%)	1.79E−02	1.20E−01	ko00500
Nitrogen metabolism	5 (1.77%)	14 (0.63%)	2.42E−02	1.42E−01	ko00910
Carbon metabolism	35 (12.41%)	205 (9.24%)	3.49E−02	1.80E−01	ko01200
Mismatch repair	5 (1.77%)	21 (0.95%)	1.18E−01	3.71E−01	ko03430
DNA replication	7 (2.48%)	34 (1.53%)	1.31E−01	3.83E−01	ko03030

## Discussion

In this study, we identified a novel CESA4 missense allele, *fc17*. Despite the defect in extension strength, *fc17* plants exhibited a relatively normally morphological phenotype compared to the WT, in contrast to previously reported *CESA4* alleles that showed significantly impaired plant growth [26, 27]. Although the *fc17* mutation caused a reduction in CESA4 protein level, the cellulose content in mature stems of the *fc17* plants only decreased by 19%, indicating that the mutant might produce enough cellulose to maintain the normal plant growth. Except for CESA, the majority of proteins involved in carbon synthesis and metabolism are upregulated in the *fc17* mutant, which might provide sufficient UDPG substrate for the production of hemicelluloses and other polysaccharides. Indeed, the *fc17* mutant exhibited increased hemicelluloses and lignin that partly complement the detrimental effect of reduced cellulose on cell wall structure. These data indicate that cell wall formation is relatively normal in the *fc17* mutant, which is further supported by the results of SEM observation. Taken together, out results indicate that the CESA4 P-CR site mutation sustains normal plant growth by producing sufficient cellulose content and by triggering the feedback response to support cell wall formation.

Notably, the *fc17* mutant has exhibited much higher plant lodging resistance than did the wild-type. Plant lodging resistance is a major and integrated agronomic trait, which is predominantly influenced by plant height and stem stiffness (breaking force) [36, 37]. Hence, the relatively short height of *fc17* might causes its enhanced lodging resistance. The *fc17* mutant displays reduced cellulose content, which provides plants with toughness, but its lignin content is significantly increased, which provides plants with excellent stiffness for resistance of biotic and abiotic stress [16, 38]. The increase of lignin content in *fc17*, therefore, is an important contributor for its high lodging resistance, which is consistent with previous studies [38]. Importantly, cellulose crystallinity has also been recently demonstrated as the main factor negatively determining plant lodging resistance in rice [10, 24]. Therefore, the *fc17* mutant showing much higher lodging resistance should be due to shorter height, increased lignin content and lower cellulose CrI.

Cellulose CrI has been considered to be a negative factor for lignocellulose digestibility because the reduced cellulose CrI increases the accessibility of the enzyme to the lignocellulose substrate [30]. Similarly, it is apparent from the data presented here that the *fc17* mutant that exhibited a reduction in CrI displayed significantly improved (30–70% increase) lignocellulose enzymatic digestibility after various chemical pretreatments compared to the WT. These results have been confirmed by observations of increased stem digestion of the *fc17* mutant in situ using SEM. Furthermore, *fc17* requires approximately three- to fourfold fewer enzymes or two-fold lower concentration of NaOH and H_2_SO_4_ to release an equal or greater amount of fermentable sugar than that in the WT plants. These data demonstrate that the *fc17* mutant will be more environmentally friendly and can be an economical option as biofuel feedstock compared with the WT.

The observed reduction in lignocellulose CrI in the *fc17* mutant may be interpreted in three ways. First, as the *fc17* mutation occurs in the P-CR region, it may affect the stability of the plasma membrane-localized CSC particles, thereby causing shorter cellulose chain lengths [19, 20, 24]. Indeed, *fc17* has exhibited a reduced cellulose DP that is negatively correlated with cellulose CrI. Second, the CESA4 subunit mutation may result in intermittent synthesis and inconsistent cellulose chain lengths, thereby disrupting the ability of neighboring cellulose chains to form crystalline structures. Third, it has been reported that hemicelluloses content negative affects cellulose CrI, and thus the increase in hemicelluloses level in the *fc17* should be an additional contributor to its lower lignocellulose CrI.

Genetic modification of plant cell walls has been considered to be a promising solution for reducing biomass recalcitrance and maintaining normal plant growth [5]. Since the transgenic approach has been used successfully, utilization of the beneficial target gene in various varieties is a more likely way to be taken. The *fc17* is a promising mutation due to its positive effects on biomass digestibility without any major adverse effects on plant growth. Based on these results, we could further introduce the mutated CESA4 into energy crops such as poplar and switchgrass to improve biomass conversion efficiency. Hence, our findings on the CESA4 P-CR region mutation may offer us a potential way for breeding energy crops.

## Conclusions

A new CESA4 allele, *fc17*, shows the substitution (F426S) mutation at the plant-conserved region of CESA4 protein. Despite reduction in cellulose content and cell wall thickness, *fc17* plants displayed relatively normal plant growth and higher lodging resistance compared to the wild-type. Multiple techniques further demonstrated that this mutation significantly reduces cellulose crystallinity, thereby enhancing biomass saccharification. Proteomic profiling based on iTRAQ assay suggested that this mutation triggers feedback response by forming a relatively integrated cell wall to maintain normal plant growth. These results suggest that the plant-conserved region of CESA4 is a promising target for genetic modification for breeding energy crop and cost-effective biomass processing.

## Methods

### Plant materials

The *fc17* was isolated from a natural population of the *Japonica* cultivar *ShenNong265*. The homozygous *fc17* mutant and wild-type (WT) plants were grown in experimental fields at Shenyang Agricultural University (Shenyang, China). The mature stem tissues were harvested and dried at 55 °C in an oven to a constant weight, and ground through 40-mesh screen (0.425 mm × 0.425 mm) and stored in a dry container until use.

### Map-based cloning of *fc17*

A 5000 F_2_ mapping population was generated from the cross between *fc17* and *MH63*, an *indica* cultivar in China. All plants were cultivated in the experimental fields at the Shenyang Agricultural University during the natural growing season. The segregation ratio in F_2_ population showed that the normal plants and Brittle Culm plants segregated as 3:1. The *fc17* gene was localized to 91-kb genomic region that contains the *CESA4* gene. The *CESA4* gene of the mutant and its corresponding WT were PCR amplified with KOD-PLUS (TOYOBO) and sequenced with a 3730 sequencer (ABI). For complementation analysis, a 8.53-kb genomic DNA fragment containing the entire *CESA4* coding region, a 1.8-kb upstream sequence, and a 1.5-kb downstream sequence were cloned into the binary vector pCAMBIA 1305 to generate the transformation plasmid. The binary plasmids were introduced into *Agrobacterium tumefaciens* strain *EHA105* and transformed into the *fc17* mutant plants.

### Measurements of plant mechanical properties and agronomic traits

The extension force of rice culms and leaves were determined using a digital force/length tester (RH-K300, Guangzhou, China). The breaking force and plant lodging index were measured at 30 days after heading as previously described [10]. Rice dry spike, dry biomass, and 1000-grain weight were respectively weighed after the samples were dried in the oven at 60 °C. All measurements were conducted using nine independent biological duplicates.

### Plant cell wall fractionation and determination

The plant cell wall fractionation procedure and total cellulose and hemicelluloses assay were conducted as previously reported [11]. The soluble sugar, lipids, and starch of the samples were successively removed from the dry biomass power samples by potassium phosphate buffer, chloroform–methanol (1:1, v/v), and DMSO-water (9:1, v/v). The remaining pellets were suspended in 4 M KOH containing 1.0 mg/mL sodium borohydride for 1 h at 25 °C, and the combined supernatants were regarded as hemicelluloses. The remaining pellets were regarded as total cellulose. All experiments were carried out in biological triplicate.

Total lignin content including acid-insoluble (AIL) and acid-soluble lignin (ASL) were detected by a two-step acid hydrolysis method as described previously [15]. Hemicelluloses monosaccharide and uronic acids (GalA and GlcA) were determined by GC–MS. The sample preparations and GC–MS analysis were conducted as previously described [11, 39].

### Immunolabeling and fluorescence imaging

The stem transverse section samples were incubated in PBS (pH 7.4) containing 5% (w/v) milk protein (MP/PBS) and a diluted antibody solution (1:50) for 1.5 h. Samples were then washed with PBS (pH 7.4) at least 3 times and incubated with a 300-fold diluted secondary antibody (GB21302, Wuhan servicebio technology Ltd., Wuhan, China) linked to fluorescein isothiocyanate (FITC) in PBS (pH 7.4) for 1.5 h in darkness.

Fluorescence was observed with a microscope equipped with epifluorescence irradiation and DIC optics (Eclipse C1, Nikon, Tokyo, Japan). Immunofluorescence (green) was observed at wavelength between 510 and 560. All micrographs were captured using equivalent settings, and representative micrographs were shown in this study.

### Cellulose CrI and DP detections

The lignocellulose crystallinity index (CrI) was detected by X-ray diffraction (XRD) method using Rigaku-D/MAX instrument (Ultima III; Japan) as previously described [12]. The relative DP of cellulose was measured by the viscometry method as described previously [12].

### Microscopic observations

The second stem internode tissues (0.5 cm sections above the node) at heading stages were cut into 1–2 mm pieces, and were observed and photographed under a scanning electron microscope (SEM TM1000, Hitachi Ltd., Tokyo, Japan).

Observation of biomass residues enzymatic digestion in vitro was conducted as previously described [11]. For plant tissue in situ enzymatic digestion, the second-stem transverse sections at heading stages were treated with 1% NaOH or 1% H_2_SO_4_, washed with distilled water until pH 7.0 and incubated with 1 g/L mixed cellulase for 2 h at 50 °C. After enzymatic hydrolysis, the tissue samples were observed and photographed under a SEM. The mixed cellulase containing β-glucanase (≥ 6 × 10^4^ U), cellulase (≥ 600 U), and xylanase (≥ 1.0 × 10^5^ U) was commercially available from Imperial Jade Bio-technology Co., Ltd (Ningxia, 750002, China).

Transmission electron microscopy (TEM) was used to observe the cell wall structures in the third leaf veins of three-leave-old seedlings as previously described [24]. The samples were washed in the PBS buffer, and then post-fixed in 2% (w/v) osmium tetroxide (OsO4) for 1 h and embedded with Super Kit (Sigma-Aldrich, St. Louis, MO, USA). Sample sections were cut with an Ultracut E ultramicrotome (Leica) and picked up on formvar-coated copper grids. After post-staining with uranyl acetate and lead citrate, the specimens were viewed under a Hitachi H7700 (Hitachi Ltd., Tokyo, Japan) transmission electron microscope.

### Biomass pretreatment and enzymatic hydrolysis

The chemical (H_2_SO_4_, NaOH) pretreatment and sequential enzymatic hydrolysis were performed as described previously [10]. For NaOH pretreatment, the ground biomass powder was added with three concentration (0.5%, 1%, 4%; w/v) of NaOH. For H_2_SO_4_ pretreatment, the biomass powder was added with three concentration (0.5%, 1%, 2%; v/v) of H_2_SO_4_ and heated at 121 °C for 20 min. The samples of chemical (NaOH, H_2_SO_4_) pretreatments were shaken at 150 r/min for 2 h at 50 °C, and centrifuged at 3000*g* for 5 min. The remaining pellet was washed with distilled water until pH 7.0. The remaining residue was collected for enzymatic hydrolysis. All experiments were performed in the biological triplicates.

### Effect of calcofluor treatment on plant growth

The germinated seeds of the *fc17* mutant and WT plants were transferred onto the MS media contained Calcofluor White dye (Sigma-Aldrich Co. LLC, California, USA) at different concentrations (0.05%, 0.1% and 0.2%). After 24-h incubation, the root length was measured every 12 h.

### Protein extraction and western blot

Fresh rice stem tissues at heading stage were ground to a fine powder in liquid nitrogen, and 0.5 g powder was extracted using Plant Total Protein Extraction Kit (Invent Biotech, Beijing, China; SD-008/SN-009) according to the manufacturer’s instructions. The protein content was measured with a BCA protein assay reagent (Beyotime Institute of Biotechnology, Jiangsu, China). The protein level of CESA4, CESA7, and CESA9 was determined by western blot analysis as described previously [24]. The dilution of anti-CESA4, anti-CESA7, anti-CESA9, and anti-tubulin (Beyotime Institute of Biotechnology, Jiangsu, China) was performed as 1:500, 1:500, 1:500, and 1:1000, respectively.

### Protein digestion

Protein digestion was performed using the FASP method [40]. A total of 300 µg proteins from each sample were placed on an ultrafiltration filter (30 kDa cutoff, Sartorius, Gottingen, Germany) that had 200 µL UA buffer (8 M urea, 150 mM Tris–HCl, pH 8.0). It was then centrifuged at 14,000*g* for 30 min and washed with 200 µL of UA buffer. About 100 µL of 50 mM iodoacetamide was added to the filter to block reduced cysteine residues. The samples were maintained at room temperature for 30 min in the dark, followed by centrifugation at a speed of 14,000*g* for 30 min. UA buffer (100 µL) was used to wash the filters twice. Approximately 100 µL of a dissolution buffer (Applied Biosystems, Foster City, CA, USA) was placed on the filter. This was centrifuged at 14,000*g* for 20 min, and then repeated twice. The protein suspensions were subjected to enzyme digestion with 40 µL of trypsin (Promega, Madison, WI, USA) buffer (4 µg trypsin in 40 µL of dissolution buffer) for 16–18 h at 37 °C. The final filter unit was transferred to a new tube that was spun at 14,000*g* for 30 min. The peptides were collected as a filtrate and the concentration of the peptides was measured at an optical density with a 280 nm wavelength (OD_280_).

### iTRAQ labeling and fractionation by strong cation exchange chromatography

Protein peptides (100 μg) from each group were labeled using the 8plex iTRAQ reagents multiplex kit (ABI, Foster City, CA, USA) (isobaric tags 113, 114, and 116 for the group without CJD and isobaric tags 118, 119, and 121 for the sCJD group). The 8plex iTRAQ reagents were allowed to reach room temperature, centrifuged, and reconstituted with 50 μL isopropyl alcohol to dissolve the iTRAQ labeling reagent. The iTRAQ labeling reagents were added to the corresponding peptide samples and reacted at room temperature for 1 h. A 100 μL aliquot of water was added to stop the labeling reaction. A 1 μL aliquot of sample was removed from each group to test labeling and extraction efficiency, and the sample was subjected to a matrix assisted laser desorption ionization procedure after Ziptip desalting. The six sample groups were pooled and vacuum-dried. Each pool of mixed peptides was lyophilized and dissolved in solution A (2% acetonitrile [ACN] and 20 mM ammonium formate, pH 10). Then, the samples were loaded onto a reverse-phase column (Luna C18, 4.6 × 150 mm; Phenomenex, Torrance, CA, USA) and eluted using a step linear elution program: 0–10% buffer B (500 mM KCl, 10 mM KH_2_PO_4_ in 25% ACN, pH 2.7) for 10 min, 10–20% buffer B for 25 min, 20–45% buffer B for 5 min, and 50–100% buffer B for 5 min at a flow rate of 0.8 mL/min. The samples were collected each min and centrifuged for 5–45 min. The fractions collected were finally combined into 10 pools and desalted on C18 Cartridges (Empore™ standard density SPE C18 Cartridges, bed I.D. 7 mm, 3 mL volume; Sigma, St. Louis, MO, USA).

### Liquid chromatography–tandem mass spectrometry (LC–MS/MS) analysis

The reconstituted peptides were analyzed with the Q-Exactive mass spectrometer (Thermo Fisher Scientific, Waltham, MA, USA) coupled with a nano high-performance liquid chromatography (UltiMate 3000 LC Dionex; Thermo Fisher Scientific) system. The peptides were loaded onto a C18-reversed phase column (3 μm-C18 resin, 75 μm × 15 cm) and separated on an analytical column (5 μm C18 resin, 150 μm × 2 cm; Dr. Maisch GmbH, Ammerbuch, Germany) using mobile phase A: 0.5% formic acid [FA]/H_2_O and B: 0.5% FA/ACN at a flow rate of 300 nL/min, using a 150 min gradient. Spectra were acquired in data-dependent mode. The 10 most intense ions selected for MS scanning (300–1800 *m*/*z*, 60,000 resolution at *m*/*z* 400, accumulation of 1 × 106 ions for a maximum of 500 ms, 1 microscan). The isolation window was 1.3 *m*/*z*, and the MS/MS spectra were accumulated for 150 ms using an Orbitrap. MS/MS spectra were measured at resolution of 15,000 at *m*/*z* 400. Dynamic precursor exclusion was allowed for 120 s after each MS/MS spectrum measurement and was set to 17,500 at *m*/*z* 200. Normalized collision energy was 30 eV and the underfill ratio, which specifies the minimum percentage of the target value likely to be reached at the maximum fill time, was defined as 0.1%. The instrument was run with peptide recognition mode enabled.

### Database search and protein quantification

The raw mass data were processed for the peptide data analysis using Proteome Discoverer 1.4 (ver. 1.4.0.288; Thermo Fisher Scientific) with a false discovery rate (FDR = *N*(decoy)*2/[(*N*(decoy) + *N*(target)] < 1% and expected cutoff or ion score < 0.05 (with 95% confidence) for searching the *Oryza sativa* UniProt database. Protein probabilities were assigned using the Protein Prophet algorithm, and proteins with at least two unique peptides were identified. The upregulated or downregulated proteins in both replicates with relative quantification *p*-values < 0.05 and 1.5-fold-changes were selected as differentially expressed.

### Bioinformatics analysis

The interactions among the differentially expressed proteins with a 1.5-fold change were determined using Pathway Studio software and the ResNet database (KEGG). The Pathway Maps tool was employed to enrich the pathways, and *p*-values were calculated based on a hypergeometric distribution, with the default database used as the background. Significant pathway enrichment was defined as a corrected FDR of *p* ≤ 0.05, and proteins with ≥ 1.5-fold change were considered differentially abundant proteins.

## Additional files

**Additional file 1.** Comparison of agronomic traits of *fc17* and its wild-type (WT) under field conditions (Shenyang, China, 2016–2017).

**Additional file 2.** 1.5-fold alterations of proteins involved in phenylpropanoid biosynthesis in comparison of *fc17* iTRAQ data to that of the WT.

**Additional file 3.** 1.5-fold alterations of proteins involved in phenylalanine metabolism in comparison of *fc17* iTRAQ data to that of the WT.

**Additional file 4.** 1.5-fold alterations of proteins involved in carbon fixation in comparison of *fc17* iTRAQ data to that of the WT.

**Additional file 5.** 1.5-fold alterations of proteins involved in carbon metabolism in comparison of *fc17* iTRAQ data to that of the WT.

**Additional file 6.** 1.5-fold alterations of proteins involved in starch and sucrose metabolism in comparison of *fc17* iTRAQ data to that of the WT.

**Additional file 7.** Comparison of proteins involved in hemicelluloses biosynthesis in the *fc17* and WT based on iTRAQ assay.

## Abbreviations

CESA: cellulose synthase
CrI: crystallinity index
CSCs: cellulose synthase complexes
CSR: class-specific region
GalA: galacturonic acid
GlcA: glucuronic acid
KEGG: Kyoto Encyclopedia of Genes and Genomes
LC–MS/MS: liquid chromatography–tandem mass spectrometry
iTRAQ: isobaric tags for relative and absolute quantification
PCWs: primary cell walls
P-CR: plant-conserved region
SCWs: secondary cell walls
SEM: scanning electron microscope
TEM: transmission electron microscopy
