# Supplementary material for: A novel FC17/CESA4 mutation causes increased biomass saccharification and lodging resistance by remodeling cell wall in rice
Source: Biotechnol Biofuels. 2018 Nov 1;11:298. doi: 10.1186/s13068-018-1298-2 (PMC6211429; doi:10.1186/s13068-018-1298-2)
Supplement: Supplementary file 1 — Additional file 1. Comparison of agronomic traits of fc17 and its wild-type (WT) under field conditions (Shenyang, China, 2016–2017). [file 13068_2018_1298_MOESM1_ESM.pptx]

## Slide 1
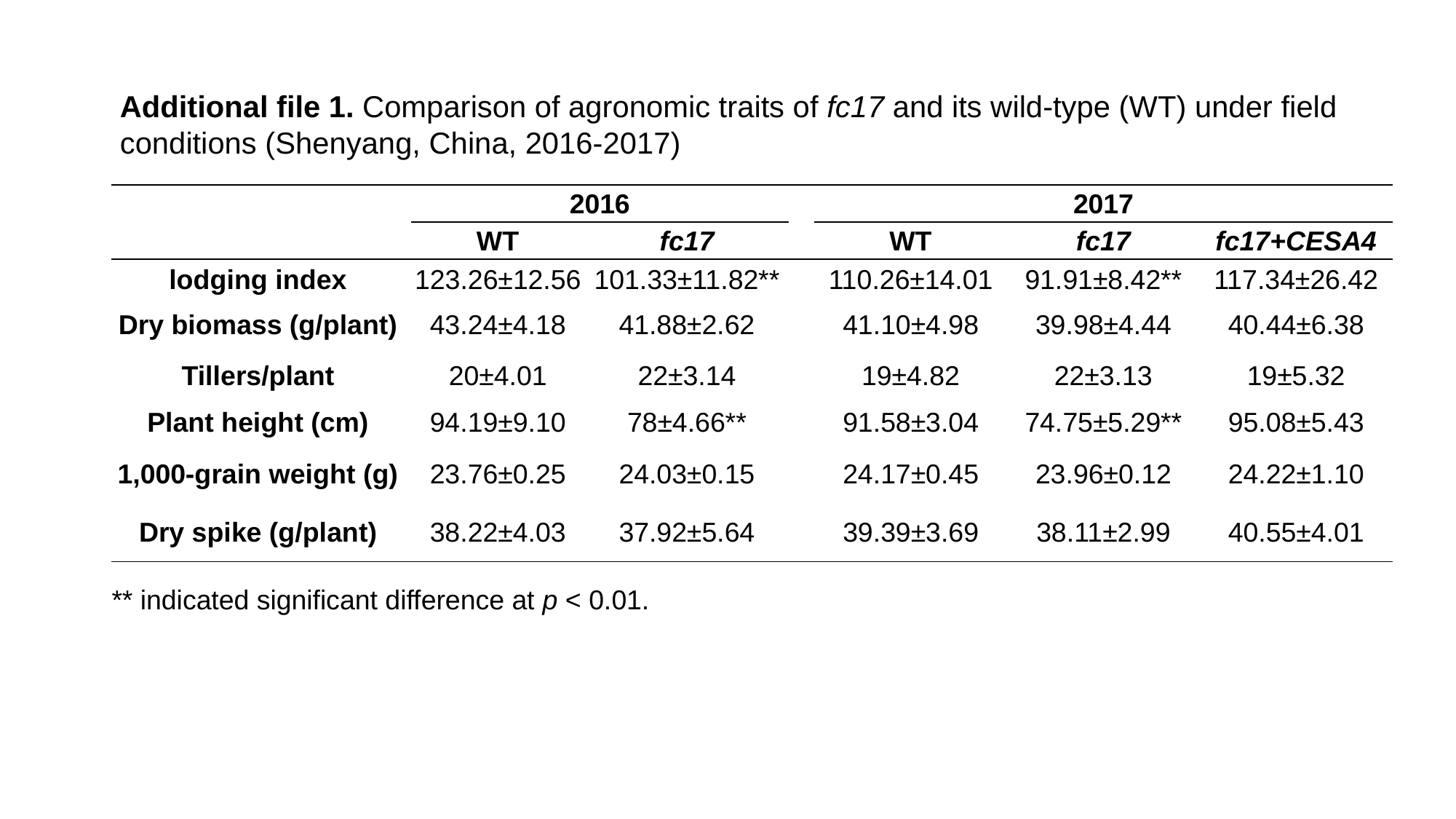

Additional file 1. Comparison of agronomic traits of fc17 and its wild-type (WT) under ﬁeld conditions (Shenyang, China, 2016-2017)
| | | 2016 | | | 2017 | | |
| --- | --- | --- | --- | --- | --- | --- | --- |
| | | WT | fc17 | | WT | fc17 | fc17+CESA4 |
| lodging index | | 123.26±12.56 | 101.33±11.82\*\* | | 110.26±14.01 | 91.91±8.42\*\* | 117.34±26.42 |
| Dry biomass (g/plant) | | 43.24±4.18 | 41.88±2.62 | | 41.10±4.98 | 39.98±4.44 | 40.44±6.38 |
| Tillers/plant | | 20±4.01 | 22±3.14 | | 19±4.82 | 22±3.13 | 19±5.32 |
| Plant height (cm) | | 94.19±9.10 | 78±4.66\*\* | | 91.58±3.04 | 74.75±5.29\*\* | 95.08±5.43 |
| 1,000-grain weight (g) | | 23.76±0.25 | 24.03±0.15 | | 24.17±0.45 | 23.96±0.12 | 24.22±1.10 |
| Dry spike (g/plant) | | 38.22±4.03 | 37.92±5.64 | | 39.39±3.69 | 38.11±2.99 | 40.55±4.01 |
** indicated significant difference at p < 0.01.
