# Supplementary material for: A novel FC17/CESA4 mutation causes increased biomass saccharification and lodging resistance by remodeling cell wall in rice
Source: Biotechnol Biofuels. 2018 Nov 1;11:298. doi: 10.1186/s13068-018-1298-2 (PMC6211429; doi:10.1186/s13068-018-1298-2)
Supplement: Supplementary file 2 — Additional file 2. 1.5-fold alterations of proteins involved in phenylpropanoid biosynthesis in comparison of fc17 iTRAQ data to that of the WT. [file 13068_2018_1298_MOESM2_ESM.pptx]

## Slide 1
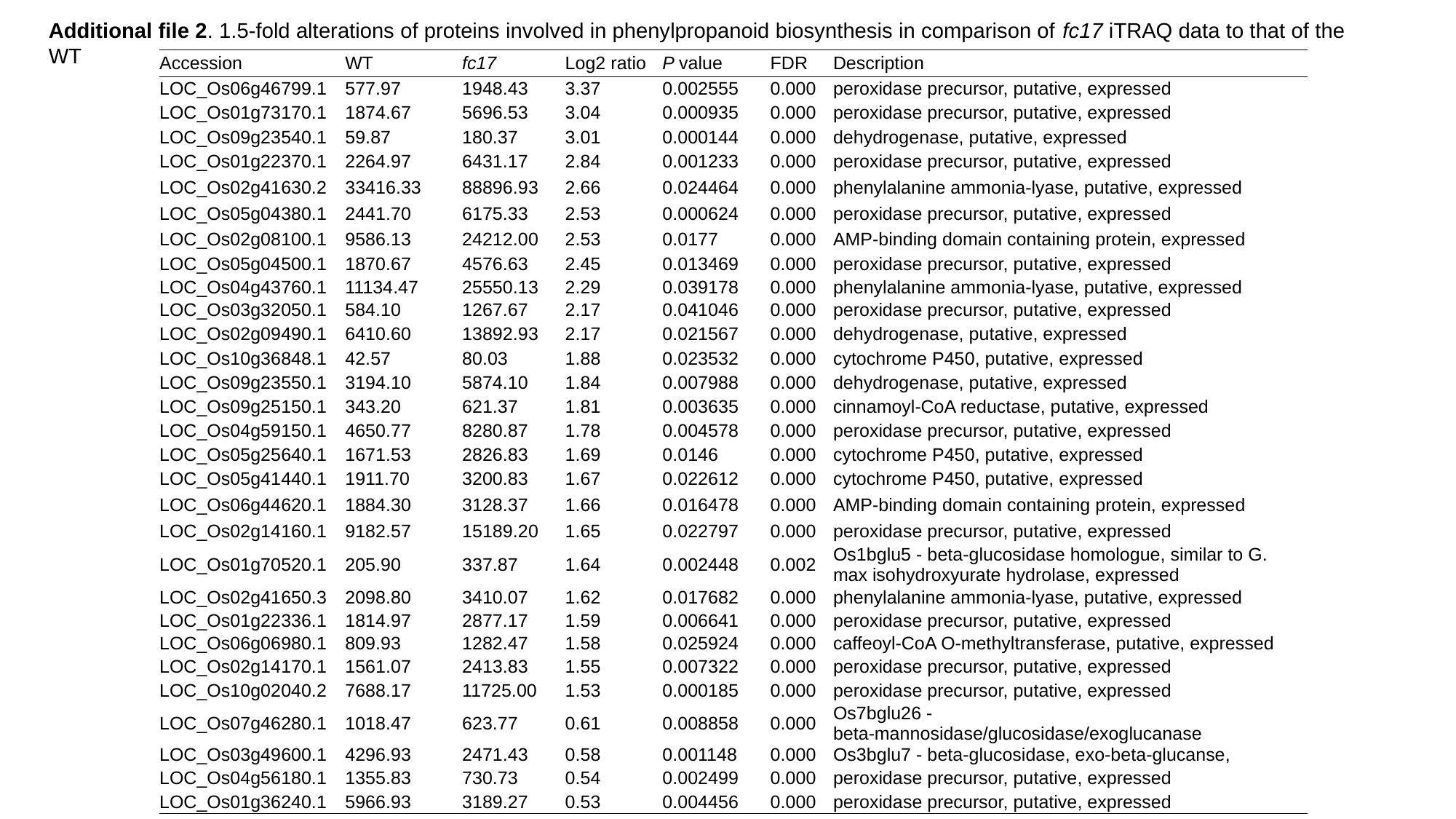

Additional file 2. 1.5-fold alterations of proteins involved in phenylpropanoid biosynthesis in comparison of fc17 iTRAQ data to that of the WT
| Accession | WT | fc17 | Log2 ratio | P value | FDR | Description |
| --- | --- | --- | --- | --- | --- | --- |
| LOC\_Os06g46799.1 | 577.97 | 1948.43 | 3.37 | 0.002555 | 0.000 | peroxidase precursor, putative, expressed |
| LOC\_Os01g73170.1 | 1874.67 | 5696.53 | 3.04 | 0.000935 | 0.000 | peroxidase precursor, putative, expressed |
| LOC\_Os09g23540.1 | 59.87 | 180.37 | 3.01 | 0.000144 | 0.000 | dehydrogenase, putative, expressed |
| LOC\_Os01g22370.1 | 2264.97 | 6431.17 | 2.84 | 0.001233 | 0.000 | peroxidase precursor, putative, expressed |
| LOC\_Os02g41630.2 | 33416.33 | 88896.93 | 2.66 | 0.024464 | 0.000 | phenylalanine ammonia-lyase, putative, expressed |
| LOC\_Os05g04380.1 | 2441.70 | 6175.33 | 2.53 | 0.000624 | 0.000 | peroxidase precursor, putative, expressed |
| LOC\_Os02g08100.1 | 9586.13 | 24212.00 | 2.53 | 0.0177 | 0.000 | AMP-binding domain containing protein, expressed |
| LOC\_Os05g04500.1 | 1870.67 | 4576.63 | 2.45 | 0.013469 | 0.000 | peroxidase precursor, putative, expressed |
| LOC\_Os04g43760.1 | 11134.47 | 25550.13 | 2.29 | 0.039178 | 0.000 | phenylalanine ammonia-lyase, putative, expressed |
| LOC\_Os03g32050.1 | 584.10 | 1267.67 | 2.17 | 0.041046 | 0.000 | peroxidase precursor, putative, expressed |
| LOC\_Os02g09490.1 | 6410.60 | 13892.93 | 2.17 | 0.021567 | 0.000 | dehydrogenase, putative, expressed |
| LOC\_Os10g36848.1 | 42.57 | 80.03 | 1.88 | 0.023532 | 0.000 | cytochrome P450, putative, expressed |
| LOC\_Os09g23550.1 | 3194.10 | 5874.10 | 1.84 | 0.007988 | 0.000 | dehydrogenase, putative, expressed |
| LOC\_Os09g25150.1 | 343.20 | 621.37 | 1.81 | 0.003635 | 0.000 | cinnamoyl-CoA reductase, putative, expressed |
| LOC\_Os04g59150.1 | 4650.77 | 8280.87 | 1.78 | 0.004578 | 0.000 | peroxidase precursor, putative, expressed |
| LOC\_Os05g25640.1 | 1671.53 | 2826.83 | 1.69 | 0.0146 | 0.000 | cytochrome P450, putative, expressed |
| LOC\_Os05g41440.1 | 1911.70 | 3200.83 | 1.67 | 0.022612 | 0.000 | cytochrome P450, putative, expressed |
| LOC\_Os06g44620.1 | 1884.30 | 3128.37 | 1.66 | 0.016478 | 0.000 | AMP-binding domain containing protein, expressed |
| LOC\_Os02g14160.1 | 9182.57 | 15189.20 | 1.65 | 0.022797 | 0.000 | peroxidase precursor, putative, expressed |
| LOC\_Os01g70520.1 | 205.90 | 337.87 | 1.64 | 0.002448 | 0.002 | Os1bglu5 - beta-glucosidase homologue, similar to G. max isohydroxyurate hydrolase, expressed |
| LOC\_Os02g41650.3 | 2098.80 | 3410.07 | 1.62 | 0.017682 | 0.000 | phenylalanine ammonia-lyase, putative, expressed |
| LOC\_Os01g22336.1 | 1814.97 | 2877.17 | 1.59 | 0.006641 | 0.000 | peroxidase precursor, putative, expressed |
| LOC\_Os06g06980.1 | 809.93 | 1282.47 | 1.58 | 0.025924 | 0.000 | caffeoyl-CoA O-methyltransferase, putative, expressed |
| LOC\_Os02g14170.1 | 1561.07 | 2413.83 | 1.55 | 0.007322 | 0.000 | peroxidase precursor, putative, expressed |
| LOC\_Os10g02040.2 | 7688.17 | 11725.00 | 1.53 | 0.000185 | 0.000 | peroxidase precursor, putative, expressed |
| LOC\_Os07g46280.1 | 1018.47 | 623.77 | 0.61 | 0.008858 | 0.000 | Os7bglu26 - beta-mannosidase/glucosidase/exoglucanase |
| LOC\_Os03g49600.1 | 4296.93 | 2471.43 | 0.58 | 0.001148 | 0.000 | Os3bglu7 - beta-glucosidase, exo-beta-glucanse, |
| LOC\_Os04g56180.1 | 1355.83 | 730.73 | 0.54 | 0.002499 | 0.000 | peroxidase precursor, putative, expressed |
| LOC\_Os01g36240.1 | 5966.93 | 3189.27 | 0.53 | 0.004456 | 0.000 | peroxidase precursor, putative, expressed |
