# Supplementary material for: A novel FC17/CESA4 mutation causes increased biomass saccharification and lodging resistance by remodeling cell wall in rice
Source: Biotechnol Biofuels. 2018 Nov 1;11:298. doi: 10.1186/s13068-018-1298-2 (PMC6211429; doi:10.1186/s13068-018-1298-2)
Supplement: Supplementary file 3 — Additional file 3. 1.5-fold alterations of proteins involved in phenylalanine metabolism in comparison of fc17 iTRAQ data to that of the WT. [file 13068_2018_1298_MOESM3_ESM.pptx]

## Slide 1
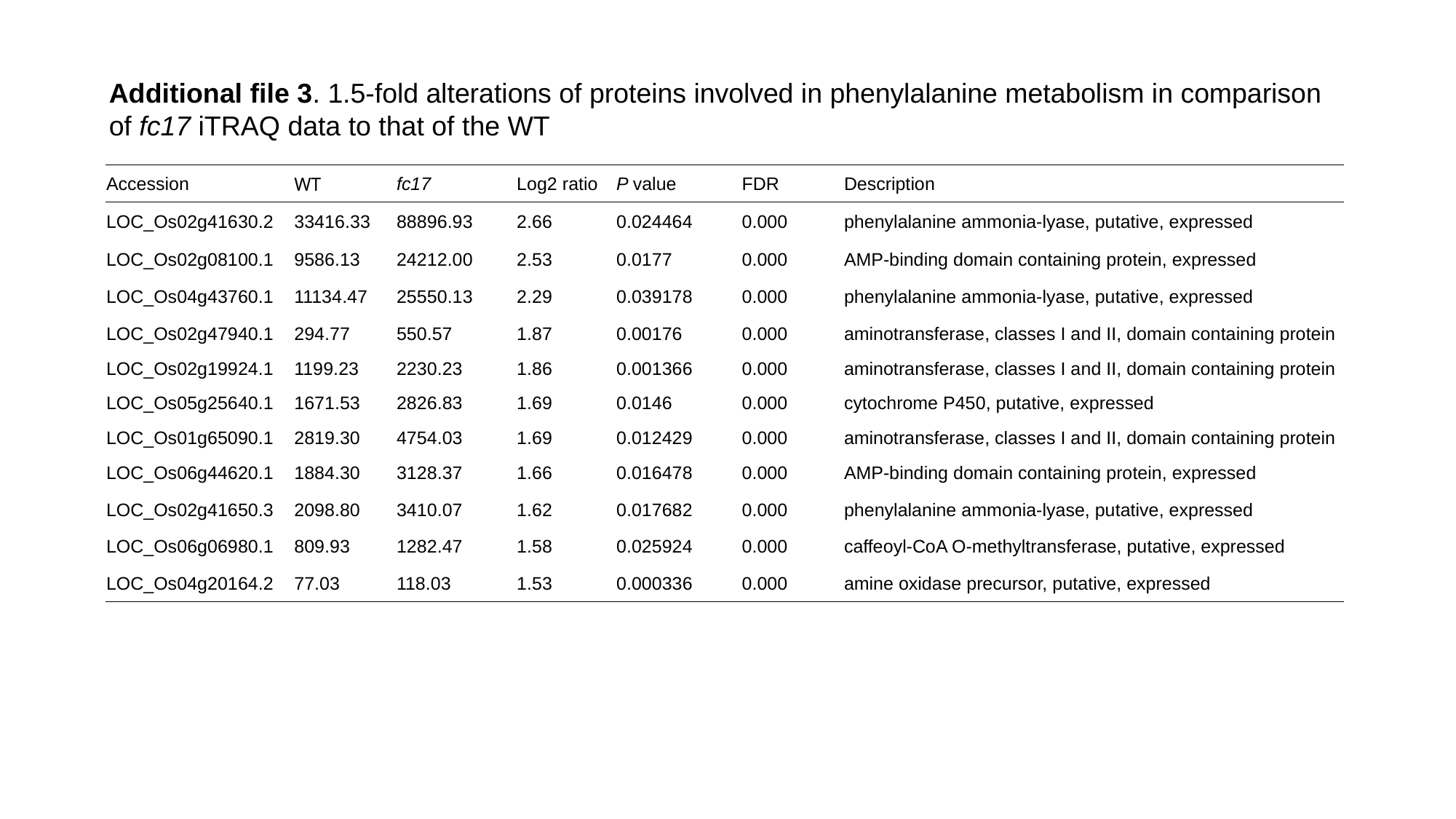

Additional file 3. 1.5-fold alterations of proteins involved in phenylalanine metabolism in comparison of fc17 iTRAQ data to that of the WT
| Accession | WT | fc17 | Log2 ratio | P value | FDR | Description |
| --- | --- | --- | --- | --- | --- | --- |
| LOC\_Os02g41630.2 | 33416.33 | 88896.93 | 2.66 | 0.024464 | 0.000 | phenylalanine ammonia-lyase, putative, expressed |
| LOC\_Os02g08100.1 | 9586.13 | 24212.00 | 2.53 | 0.0177 | 0.000 | AMP-binding domain containing protein, expressed |
| LOC\_Os04g43760.1 | 11134.47 | 25550.13 | 2.29 | 0.039178 | 0.000 | phenylalanine ammonia-lyase, putative, expressed |
| LOC\_Os02g47940.1 | 294.77 | 550.57 | 1.87 | 0.00176 | 0.000 | aminotransferase, classes I and II, domain containing protein |
| LOC\_Os02g19924.1 | 1199.23 | 2230.23 | 1.86 | 0.001366 | 0.000 | aminotransferase, classes I and II, domain containing protein |
| LOC\_Os05g25640.1 | 1671.53 | 2826.83 | 1.69 | 0.0146 | 0.000 | cytochrome P450, putative, expressed |
| LOC\_Os01g65090.1 | 2819.30 | 4754.03 | 1.69 | 0.012429 | 0.000 | aminotransferase, classes I and II, domain containing protein |
| LOC\_Os06g44620.1 | 1884.30 | 3128.37 | 1.66 | 0.016478 | 0.000 | AMP-binding domain containing protein, expressed |
| LOC\_Os02g41650.3 | 2098.80 | 3410.07 | 1.62 | 0.017682 | 0.000 | phenylalanine ammonia-lyase, putative, expressed |
| LOC\_Os06g06980.1 | 809.93 | 1282.47 | 1.58 | 0.025924 | 0.000 | caffeoyl-CoA O-methyltransferase, putative, expressed |
| LOC\_Os04g20164.2 | 77.03 | 118.03 | 1.53 | 0.000336 | 0.000 | amine oxidase precursor, putative, expressed |
