# Supplementary material for: A novel FC17/CESA4 mutation causes increased biomass saccharification and lodging resistance by remodeling cell wall in rice
Source: Biotechnol Biofuels. 2018 Nov 1;11:298. doi: 10.1186/s13068-018-1298-2 (PMC6211429; doi:10.1186/s13068-018-1298-2)
Supplement: Supplementary file 5 — Additional file 5. 1.5-fold alterations of proteins involved in carbon metabolism in comparison of fc17 iTRAQ data to that of the WT. [file 13068_2018_1298_MOESM5_ESM.pptx]

## Slide 1
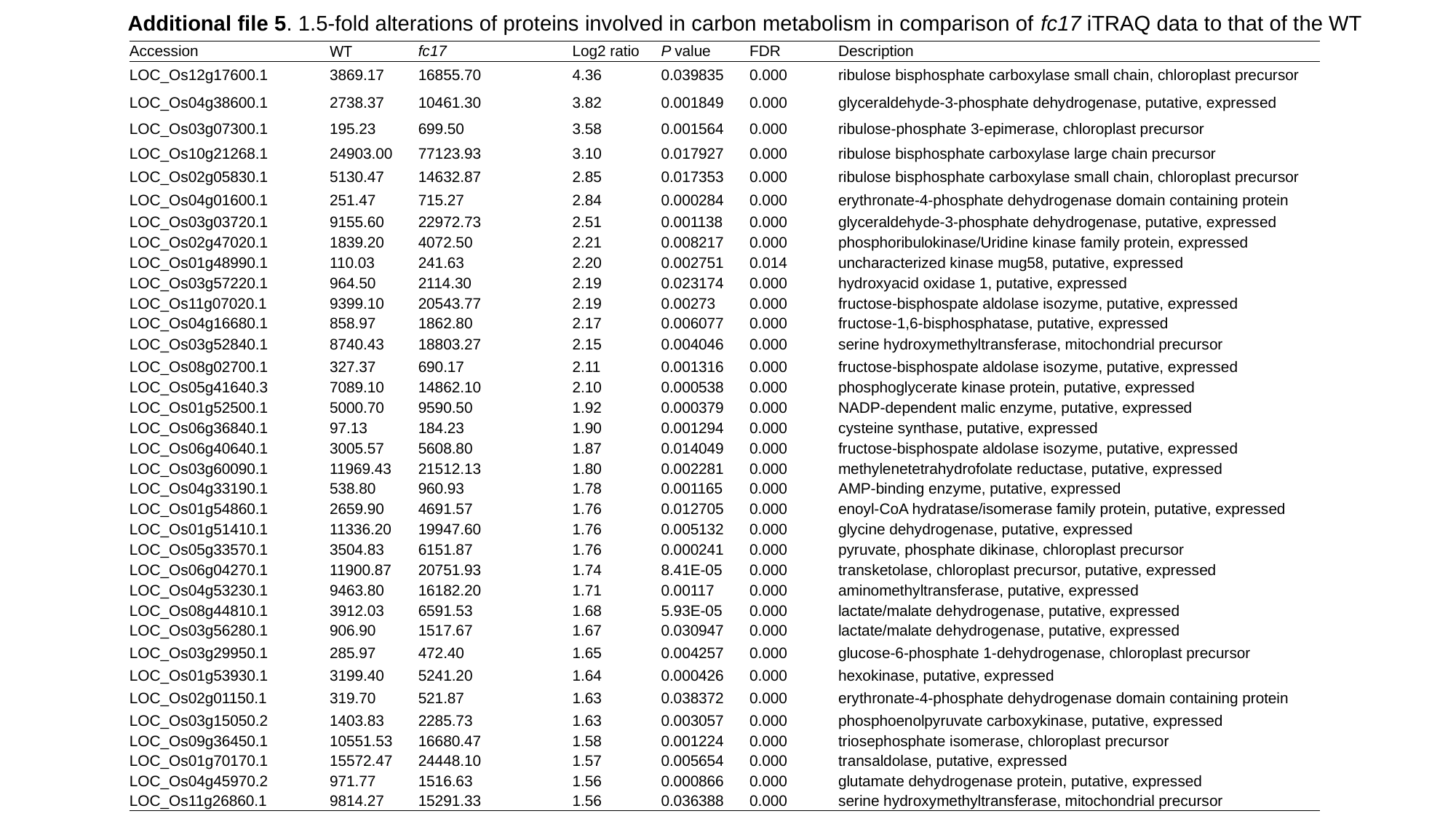

Additional file 5. 1.5-fold alterations of proteins involved in carbon metabolism in comparison of fc17 iTRAQ data to that of the WT
| Accession | WT | fc17 | Log2 ratio | P value | FDR | Description |
| --- | --- | --- | --- | --- | --- | --- |
| LOC\_Os12g17600.1 | 3869.17 | 16855.70 | 4.36 | 0.039835 | 0.000 | ribulose bisphosphate carboxylase small chain, chloroplast precursor |
| LOC\_Os04g38600.1 | 2738.37 | 10461.30 | 3.82 | 0.001849 | 0.000 | glyceraldehyde-3-phosphate dehydrogenase, putative, expressed |
| LOC\_Os03g07300.1 | 195.23 | 699.50 | 3.58 | 0.001564 | 0.000 | ribulose-phosphate 3-epimerase, chloroplast precursor |
| LOC\_Os10g21268.1 | 24903.00 | 77123.93 | 3.10 | 0.017927 | 0.000 | ribulose bisphosphate carboxylase large chain precursor |
| LOC\_Os02g05830.1 | 5130.47 | 14632.87 | 2.85 | 0.017353 | 0.000 | ribulose bisphosphate carboxylase small chain, chloroplast precursor |
| LOC\_Os04g01600.1 | 251.47 | 715.27 | 2.84 | 0.000284 | 0.000 | erythronate-4-phosphate dehydrogenase domain containing protein |
| LOC\_Os03g03720.1 | 9155.60 | 22972.73 | 2.51 | 0.001138 | 0.000 | glyceraldehyde-3-phosphate dehydrogenase, putative, expressed |
| LOC\_Os02g47020.1 | 1839.20 | 4072.50 | 2.21 | 0.008217 | 0.000 | phosphoribulokinase/Uridine kinase family protein, expressed |
| LOC\_Os01g48990.1 | 110.03 | 241.63 | 2.20 | 0.002751 | 0.014 | uncharacterized kinase mug58, putative, expressed |
| LOC\_Os03g57220.1 | 964.50 | 2114.30 | 2.19 | 0.023174 | 0.000 | hydroxyacid oxidase 1, putative, expressed |
| LOC\_Os11g07020.1 | 9399.10 | 20543.77 | 2.19 | 0.00273 | 0.000 | fructose-bisphospate aldolase isozyme, putative, expressed |
| LOC\_Os04g16680.1 | 858.97 | 1862.80 | 2.17 | 0.006077 | 0.000 | fructose-1,6-bisphosphatase, putative, expressed |
| LOC\_Os03g52840.1 | 8740.43 | 18803.27 | 2.15 | 0.004046 | 0.000 | serine hydroxymethyltransferase, mitochondrial precursor |
| LOC\_Os08g02700.1 | 327.37 | 690.17 | 2.11 | 0.001316 | 0.000 | fructose-bisphospate aldolase isozyme, putative, expressed |
| LOC\_Os05g41640.3 | 7089.10 | 14862.10 | 2.10 | 0.000538 | 0.000 | phosphoglycerate kinase protein, putative, expressed |
| LOC\_Os01g52500.1 | 5000.70 | 9590.50 | 1.92 | 0.000379 | 0.000 | NADP-dependent malic enzyme, putative, expressed |
| LOC\_Os06g36840.1 | 97.13 | 184.23 | 1.90 | 0.001294 | 0.000 | cysteine synthase, putative, expressed |
| LOC\_Os06g40640.1 | 3005.57 | 5608.80 | 1.87 | 0.014049 | 0.000 | fructose-bisphospate aldolase isozyme, putative, expressed |
| LOC\_Os03g60090.1 | 11969.43 | 21512.13 | 1.80 | 0.002281 | 0.000 | methylenetetrahydrofolate reductase, putative, expressed |
| LOC\_Os04g33190.1 | 538.80 | 960.93 | 1.78 | 0.001165 | 0.000 | AMP-binding enzyme, putative, expressed |
| LOC\_Os01g54860.1 | 2659.90 | 4691.57 | 1.76 | 0.012705 | 0.000 | enoyl-CoA hydratase/isomerase family protein, putative, expressed |
| LOC\_Os01g51410.1 | 11336.20 | 19947.60 | 1.76 | 0.005132 | 0.000 | glycine dehydrogenase, putative, expressed |
| LOC\_Os05g33570.1 | 3504.83 | 6151.87 | 1.76 | 0.000241 | 0.000 | pyruvate, phosphate dikinase, chloroplast precursor |
| LOC\_Os06g04270.1 | 11900.87 | 20751.93 | 1.74 | 8.41E-05 | 0.000 | transketolase, chloroplast precursor, putative, expressed |
| LOC\_Os04g53230.1 | 9463.80 | 16182.20 | 1.71 | 0.00117 | 0.000 | aminomethyltransferase, putative, expressed |
| LOC\_Os08g44810.1 | 3912.03 | 6591.53 | 1.68 | 5.93E-05 | 0.000 | lactate/malate dehydrogenase, putative, expressed |
| LOC\_Os03g56280.1 | 906.90 | 1517.67 | 1.67 | 0.030947 | 0.000 | lactate/malate dehydrogenase, putative, expressed |
| LOC\_Os03g29950.1 | 285.97 | 472.40 | 1.65 | 0.004257 | 0.000 | glucose-6-phosphate 1-dehydrogenase, chloroplast precursor |
| LOC\_Os01g53930.1 | 3199.40 | 5241.20 | 1.64 | 0.000426 | 0.000 | hexokinase, putative, expressed |
| LOC\_Os02g01150.1 | 319.70 | 521.87 | 1.63 | 0.038372 | 0.000 | erythronate-4-phosphate dehydrogenase domain containing protein |
| LOC\_Os03g15050.2 | 1403.83 | 2285.73 | 1.63 | 0.003057 | 0.000 | phosphoenolpyruvate carboxykinase, putative, expressed |
| LOC\_Os09g36450.1 | 10551.53 | 16680.47 | 1.58 | 0.001224 | 0.000 | triosephosphate isomerase, chloroplast precursor |
| LOC\_Os01g70170.1 | 15572.47 | 24448.10 | 1.57 | 0.005654 | 0.000 | transaldolase, putative, expressed |
| LOC\_Os04g45970.2 | 971.77 | 1516.63 | 1.56 | 0.000866 | 0.000 | glutamate dehydrogenase protein, putative, expressed |
| LOC\_Os11g26860.1 | 9814.27 | 15291.33 | 1.56 | 0.036388 | 0.000 | serine hydroxymethyltransferase, mitochondrial precursor |
