# Supplementary material for: A novel FC17/CESA4 mutation causes increased biomass saccharification and lodging resistance by remodeling cell wall in rice
Source: Biotechnol Biofuels. 2018 Nov 1;11:298. doi: 10.1186/s13068-018-1298-2 (PMC6211429; doi:10.1186/s13068-018-1298-2)
Supplement: Supplementary file 6 — Additional file 6. 1.5-fold alterations of proteins involved in starch and sucrose metabolism in comparison of fc17 iTRAQ data to that of the WT. [file 13068_2018_1298_MOESM6_ESM.pptx]

## Slide 1
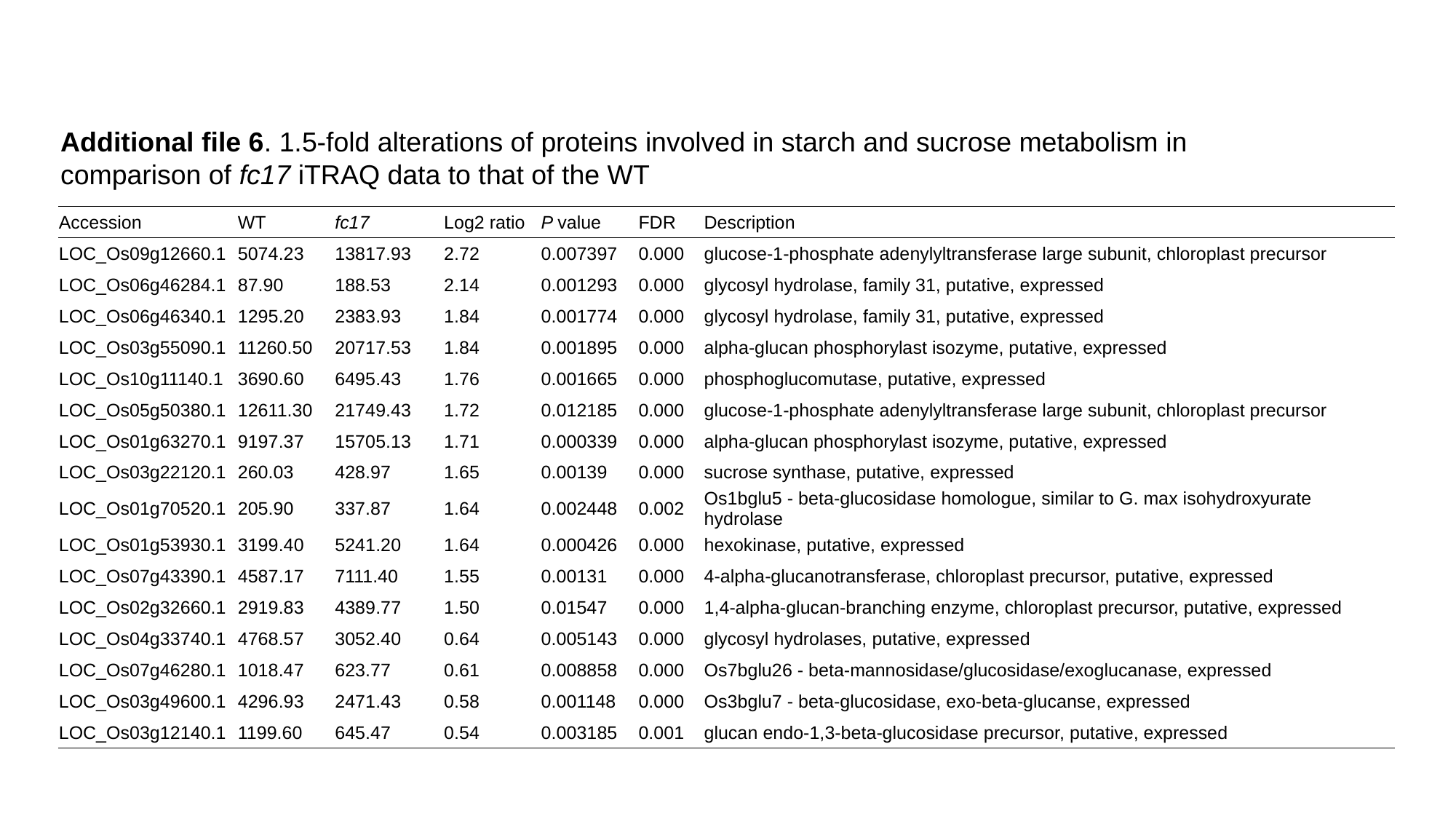

Additional file 6. 1.5-fold alterations of proteins involved in starch and sucrose metabolism in comparison of fc17 iTRAQ data to that of the WT
| Accession | WT | fc17 | Log2 ratio | P value | FDR | Description |
| --- | --- | --- | --- | --- | --- | --- |
| LOC\_Os09g12660.1 | 5074.23 | 13817.93 | 2.72 | 0.007397 | 0.000 | glucose-1-phosphate adenylyltransferase large subunit, chloroplast precursor |
| LOC\_Os06g46284.1 | 87.90 | 188.53 | 2.14 | 0.001293 | 0.000 | glycosyl hydrolase, family 31, putative, expressed |
| LOC\_Os06g46340.1 | 1295.20 | 2383.93 | 1.84 | 0.001774 | 0.000 | glycosyl hydrolase, family 31, putative, expressed |
| LOC\_Os03g55090.1 | 11260.50 | 20717.53 | 1.84 | 0.001895 | 0.000 | alpha-glucan phosphorylast isozyme, putative, expressed |
| LOC\_Os10g11140.1 | 3690.60 | 6495.43 | 1.76 | 0.001665 | 0.000 | phosphoglucomutase, putative, expressed |
| LOC\_Os05g50380.1 | 12611.30 | 21749.43 | 1.72 | 0.012185 | 0.000 | glucose-1-phosphate adenylyltransferase large subunit, chloroplast precursor |
| LOC\_Os01g63270.1 | 9197.37 | 15705.13 | 1.71 | 0.000339 | 0.000 | alpha-glucan phosphorylast isozyme, putative, expressed |
| LOC\_Os03g22120.1 | 260.03 | 428.97 | 1.65 | 0.00139 | 0.000 | sucrose synthase, putative, expressed |
| LOC\_Os01g70520.1 | 205.90 | 337.87 | 1.64 | 0.002448 | 0.002 | Os1bglu5 - beta-glucosidase homologue, similar to G. max isohydroxyurate hydrolase |
| LOC\_Os01g53930.1 | 3199.40 | 5241.20 | 1.64 | 0.000426 | 0.000 | hexokinase, putative, expressed |
| LOC\_Os07g43390.1 | 4587.17 | 7111.40 | 1.55 | 0.00131 | 0.000 | 4-alpha-glucanotransferase, chloroplast precursor, putative, expressed |
| LOC\_Os02g32660.1 | 2919.83 | 4389.77 | 1.50 | 0.01547 | 0.000 | 1,4-alpha-glucan-branching enzyme, chloroplast precursor, putative, expressed |
| LOC\_Os04g33740.1 | 4768.57 | 3052.40 | 0.64 | 0.005143 | 0.000 | glycosyl hydrolases, putative, expressed |
| LOC\_Os07g46280.1 | 1018.47 | 623.77 | 0.61 | 0.008858 | 0.000 | Os7bglu26 - beta-mannosidase/glucosidase/exoglucanase, expressed |
| LOC\_Os03g49600.1 | 4296.93 | 2471.43 | 0.58 | 0.001148 | 0.000 | Os3bglu7 - beta-glucosidase, exo-beta-glucanse, expressed |
| LOC\_Os03g12140.1 | 1199.60 | 645.47 | 0.54 | 0.003185 | 0.001 | glucan endo-1,3-beta-glucosidase precursor, putative, expressed |
