# Supplementary material for: A novel FC17/CESA4 mutation causes increased biomass saccharification and lodging resistance by remodeling cell wall in rice
Source: Biotechnol Biofuels. 2018 Nov 1;11:298. doi: 10.1186/s13068-018-1298-2 (PMC6211429; doi:10.1186/s13068-018-1298-2)
Supplement: Supplementary file 7 — Additional file 7. Comparison of proteins involved in hemicelluloses biosynthesis in the fc17 and WT based on iTRAQ assay. [file 13068_2018_1298_MOESM7_ESM.pptx]

## Slide 1
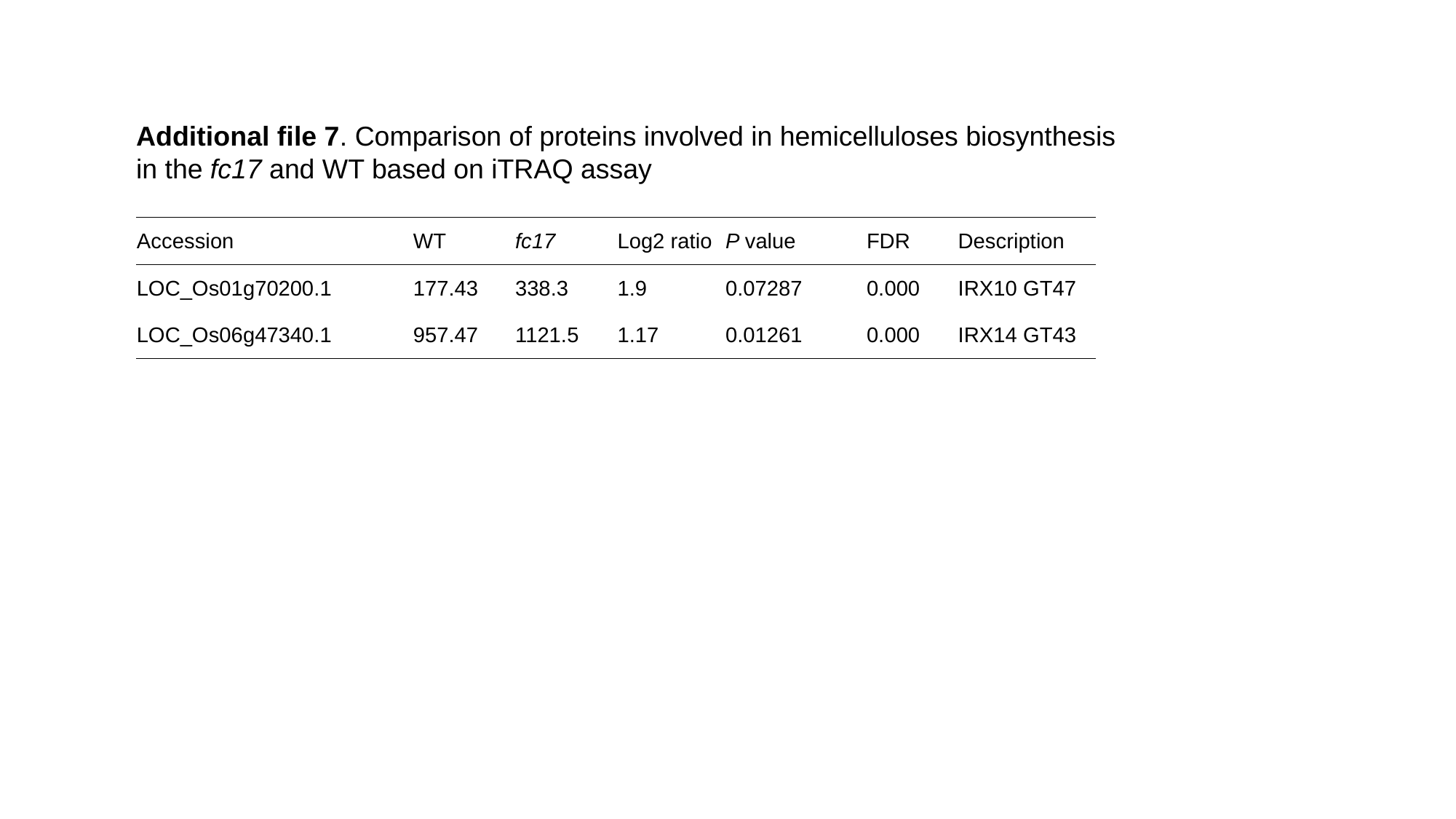

Additional file 7. Comparison of proteins involved in hemicelluloses biosynthesis in the fc17 and WT based on iTRAQ assay
| Accession | WT | fc17 | Log2 ratio | P value | FDR | Description |
| --- | --- | --- | --- | --- | --- | --- |
| LOC\_Os01g70200.1 | 177.43 | 338.3 | 1.9 | 0.07287 | 0.000 | IRX10 GT47 |
| LOC\_Os06g47340.1 | 957.47 | 1121.5 | 1.17 | 0.01261 | 0.000 | IRX14 GT43 |
